# Self‐leadership and why it matters to nurses: A scoping review

**DOI:** 10.1111/inr.70014

**Published:** 2025-03-05

**Authors:** Katja Pursio, Tarja Kvist, Päivi Kankkunen, Laura A. Fennimore

**Affiliations:** ^1^ Department of Nursing Science University of Eastern Finland Kuopio Finland; ^2^ School of Nursing University of Pittsburgh Pittsburgh Pennsylvania USA

**Keywords:** hospital, nurse, scoping review, self‐leadership, self‐leadership skills, synthesis

## Abstract

**Aim:**

To synthesize the previous findings of nurses’ self‐leadership in hospital settings.

**Background:**

Self‐leadership is a process of comprehensive self‐influence. People direct themselves toward optimal performance with self‐motivation and take responsibility for their actions. Self‐leadership has been found to have a connection with the experience of meaningfulness of work, commitment, and job satisfaction.

**Methods:**

The scoping review was conducted by following the PRISMA‐ScR reporting checklist. PubMed, CINAHL, Scopus, and PsycINFO databases were searched, and 1831 articles were identified and screened. Multiple study designs were included while specific inclusion and exclusion criteria regarding population, concept, and context were addressed. Narrative data synthesis was conducted.

**Findings:**

The search identified a total of 13 relevant studies published between 2013 and 2023. Four themes were found to describe nurses’ self‐leadership: Self‐leadership is nurses' internal skill that increases with experience (*n* = 8); self‐leadership improves nurses' work performance (*n* = 8); self‐leadership supports work well‐being (*n* = 5); and self‐leadership thrives in favorable nursing work environments (*n* = 6).

**Conclusion:**

Self‐leadership has a positive connection to nurses’ work performance and well‐being. Nursing students should be introduced to self‐leadership skill development in their nursing education programs, and nurses should be offered continuing education opportunities to develop self‐leadership skills throughout their careers. Further studies are necessary to fill the information gap about explaining self‐leadership in the context of professional nursing and offering recommendations for how to strengthen nurses’ self‐leadership skills.

**Implications for nursing education, practice, and policy:**

Self‐leadership encourages nurses to work proactively to improve patient care and enhance work environments. Self‐leadership includes behaviors that can be encouraged through each developmental stage of a nurse's career. Faculty, nurse leaders, and organizational structures play an important role in identifying self‐leadership behaviors and supporting their positive development.

## INTRODUCTION

Nursing care is delivered worldwide in complex healthcare systems that are challenged by workforce shortages, aging and diverse populations, and an explosion of new technology and medical advances (National Academies of Sciences, Engineering, & Medicine, 2021). Hospital nurse leaders are confronted with increased needs for their time and attention due to expanding spans of control, regulatory demands, and organizational needs to provide quality patient care (Zangerle & Martin, 2024). Nurse leaders must share accountability for these goals with their teams and encourage self‐leadership behaviors among staff nurses (Knotts et al., 2021; Liu et al., 2023). Nurses demonstrate self‐leadership by developing their knowledge, skills, and attitudes related to clinical practice to improve their performance and competence.

Self‐leadership is closely related to the concept of shared leadership, in which a formal leader, such as a nurse manager, shares traditional leadership roles and responsibilities with staff through coaching and empowerment activities (Currie & Spyridonidis, 2019; Olender et al., 2020). The positive effects of shared leadership on nurses’ empowerment and engagement have been well documented in the literature (Porter‐O'Grady & Clavelle, 2021). However, shared leadership is not possible unless employees take responsibility for their participation and self‐leadership (Olender et al., 2020). Porter‐O'Grady and Clavelle (2021) raised concerns that nurses do not know what their role is in shared leadership or how they can take ownership of their own work. Understanding the context of self‐leadership is essential to exploring this nuanced concept.

## CONCEPTUAL MODELS

Conceptual models of self‐leadership are based on multiple behavioral theories related to self‐determination, social cognition, self‐regulation, self‐management, and positive psychology. Self‐management and self‐leadership are very similar concepts; the key difference is that self‐leadership addresses what, why, and how something should be done, whereas self‐management addresses only how it should be done (Neck et al., 2019).

Self‐leadership strategies are usually separated into three categories based on their focus on behavior, natural rewards, and thoughts. Self‐imposed *behavior‐focused strategies* strive to encourage positive behaviors that lead to desired outcomes. These strategies include self‐observation, self‐goal setting, self‐reward, self‐punishment, self‐cueing, and practice. *Natural reward strategies* help to create feelings of competence, self‐control, and purpose. Natural reward strategies build more pleasant features into a given task so that the task itself becomes naturally rewarding or draws attention away from the less pleasant components of the activity. Unpleasant aspects of work can vary per employee and can be related to conditions that cannot be influenced. Finally, *constructive thought pattern strategies* focus on positive thinking, including positive self‐talk, evaluation of beliefs and assumptions, and mental practice, which means creating an imagination about the success of the upcoming event (Neck et al., 2019).

Organizational citizenship behaviors, such as conscientiousness, loyalty, sportsmanship, courtesy, and helpfulness toward other people, impact goal achievement as individuals feel psychologically empowered to make a difference within their organization. A study of 261 nurses explored the impact of psychological empowerment on individual‐ and organization‐related citizenship behaviors. A positive correlation (*p* < 0.01) was noted across all dimensions at both the individual and organization levels for impact (*r* = 0.56; *r* = 0.60), meaning (0.51, 0.53), self‐determination (*r* = 0.53, *r* = 0.57), and competence (*r* = 0.50, *r* = 0.52) (Islam & Ifran, 2022).

“Every nurse is a leader” is a frequent statement often quoted in the context of clinical leadership; however, not all clinical nurses at the bedside see themselves as leaders (Booher et al., 2021). Previous studies have focused on nurses’ clinical leadership (Booher et al., 2021; Maenhout et al., 2021), which can be confused with the concept of self‐leadership. Clinical leadership focuses on patients and includes coordination of patient care, collaboration with other members of the healthcare team using effective communication skills, and leading patients toward health and wellness (Booher et al., 2021). Self‐leadership is an internal process in which employees use intrinsic motivational, cognitive, and behavioral strategies to lead themselves (Stewart et al., 2011).

Nurse leaders can shape staff nurses’ self‐leadership by modeling these behaviors through leadership styles that promote competency development and interprofessional collaboration. A systematic review noted the positive impact of nurse leader style on staff nurses’ performance, including servant (humanistic and humble), transformational, and transactional, collaborative, paternalistic, and entrepreneurial leadership (Alsadaan et al., 2023). Nurses should be supported to increase their self‐leadership by their superiors and receive training to build their self‐leadership skills (Pursio et al., 2023).

The International Council of Nurses (ICN) has a long tradition of preparing nurses across the globe to serve as effective leaders and impact health policy through the ICN Leadership for Change™ program and the ICN Global Nursing Leadership Institute (Ferguson et al., 2016; Mason & Salvage, 2021). A comprehensive understanding of what is meant by nursing self‐leadership is needed, both as a concept and in practice, to establish professional development programs for staff nurses. This scoping review sought to answer the research question “what factors impact nurses’ self‐leadership behaviors in hospital settings?”

## METHODS

### Design

The scoping review method was chosen due to its exploratory and descriptive nature. This study aimed to ensure an extensive overview of nurses’ self‐leadership by systematically mapping the existing literature and identifying research gaps (Munn et al., 2022; Peters et al., 2020). The review was conducted following updated methodological guidance for scoping reviews (Peters et al., 2020) and the PRISMA‐ScR reporting checklist (Tricco et al., 2018).

### Search method

The search strategy was based on the PCC mnemonic (population–concept–context). The key terms were designed, tested, and refined in collaboration with an information specialist. Combinations of relevant search terms were formed using Boolean terms AND OR, and the final search strategy was as follows: nurse AND self‐leadership OR self‐concept OR self‐regulation OR self‐actualization OR self‐determination AND hospital. PubMed, CINAHL, Scopus, and PsycINFO databases were searched for articles published in English between 2013 and 2023 (Supporting Information File S1).

**FIGURE 1:**
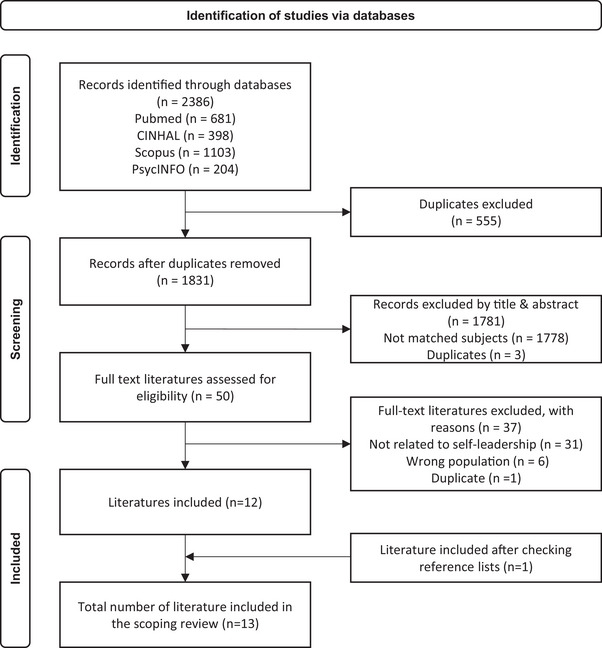
Flow diagram according to the PRISMA 2020 statement (Page et al., 2021).

The initial database search was conducted by the first author, and the search outcomes from all databases were transformed into a web‐based platform in Covidence. Duplicates were removed automatically. In Covidence, each result was screened by two researchers in two phases: first the title and abstract, and then the full text. Conflicts regarding the relevance of the results were resolved through joint discussion. The screening process is illustrated in Figure 1.

### Inclusion and exclusion criteria

Multiple study designs were included in this scoping review. To form a coherent body of research on self‐leadership, we searched for studies that focused on clinical nurses working in hospitals with similar nursing education and scope of practice. The inclusion criteria were studies regarding licensed nurses (staff nurses, registered nurses, and advanced nurse roles such as nurse practitioners, nurse specialists, and nurse educators) and outcomes related to self‐leadership, self‐leadership skills, self‐determination, self‐actualization, or self‐concept. Studies were excluded if they focused on nonlicensed caregivers, nursing students, managers and leaders, or other healthcare professionals. Studies were excluded from the review if they explored outcomes related to nurses’ clinical leadership or patient self‐management, or if they addressed practice outside of hospitals or clinical settings.

### Search outcomes

A total of 2386 articles were identified, and after removing duplicates, 1831 articles were screened with a review of the title and abstract. Next, 1778 articles were excluded due to a mismatch with the inclusion criteria, and three additional duplicates were identified. Fifty full‐text articles were screened and assessed for their eligibility. Twelve articles were deemed relevant by the reviewers. The first author screened the reference lists of the included papers and found one additional match that was accepted for inclusion in the scoping review with mutual consent of the authors. Thirteen studies were included in this review (Figure 1).

### Data extraction

A data table was developed to document the data extracted from each study including authors, study aims and design, sample, setting, relevant findings in line with the research questions, and a quality assessment identifying strengths and limitations of studies to strengthen transparency and clarity of evidence (Table 1). The first author manually extracted data from the studies and compiled the main points in a table, which was revised by the research team to reduce the chance of bias and errors (Peters et al., 2020).

**TABLE 1:** Aim, design, participants, and findings of each of the 13 studies included in the review.

Author	Aim	Design	Participants	Findings	Strengths and limitations
Alabdulbaq et al. (2019), Saudi Arabia	To explore the relationship between self‐leadership and emotional intelligence among nurses.	Cross‐sectional study.	158 nurses from one hospital in Saudi Arabia.	Most of the nurses assessed their self‐leadership to a high level (M 32.6; SD 8.6), as well as 74.1% of nurses had high emotional intelligence (M 59.4; SD 13.4). Moreover, there was a statistically significant relationship between self‐leadership and emotional intelligence (*p* = 0.016). Nurses’ demographic characteristics did not show a significant difference in the level of self‐leadership while education (diploma vs bachelor) showed nearly statistical significance (OR 2.267; *p* < 0.055). Instead, nationality (Saudi vs. Non‐Saudi) showed significant difference in the level of emotional intelligence (OR 5.027; *p* = 0.004).	Strengths: ‐ Study subjects, settings, and data collection are described in detail ‐ The adequacy of the sample size for statistical analysis is justified Limitations: ‐ Low sample size, which has not been discussed ‐ Authors themselves did not highlight any limitations of the study
Chen and Zhou (2022), China	To analyze the impact of nurses' self‐leadership on peer voice endorsement through thriving at work. In addition, to introduce task conflict and relationship conflict as mediators.	Cross‐sectional study.	405 nurses from one hospital in China.	Nurses assessed their self‐leadership as 5.89 (SD 0.81) out of 7. Nurses’ self‐leadership had a positive effect on thriving at work (B 0.24; *p* < 0.001), and peer voice endorsement through the mediating effect of thriving at work (B 0.103; *p* < 0.05).	Strengths: ‐ The data collection is described in detail ‐ Large sample size ‐ The study measures outcome and antecedent variables separately ‐ The practical implications of the findings are presented concretely and from many perspectives Limitations: ‐ Study subjects and settings have not been described
Gomes et al. (2015), Portugal	To explore the existence of different profiles of self‐leadership strategies, and test if they have effects on nurses’ ability to be innovative.	Cross‐sectional study.	288 nurses from various healthcare units in Portugal.	The nurses agreed in applying strategies like self‐goal setting (M 5.92; SD 0.9), self‐talk (M 5.51; SD 1.11), natural reward strategies (M 5.47; SD 0.95), self‐evaluation of one's values and beliefs (M 4.93; SD 1.06) and visualizing successful performance (M 5.00; SD 1.09). Instead, the nurses neither agreed nor disagreed in applying strategies like self‐reward (M 4.01; SD 1.52). Nurses also indicated that they sometimes introduced new methodologies (M 3. 19; SD 0.72), and improved ways to perform tasks at work. A 3‐cluster solution, where nurses who used only some strategies of self‐leadership, explained the data best.	Strengths: ‐ Nurses represented different units from different specialties ‐ Study presents three different clusters of self‐leadership ‐ The practical implications of the findings are presented concretely and from many perspectives Limitations: ‐ Details of the respondents' inclusion and exclusion criteria have not been described ‐ Data collection has not been reported
Huang and Zhou (2023), China	To explore how the interaction of self‐sacrificial leadership and self‐leadership is related to thriving at work, work well‐being, and work–family conflict during the COVID‐19 pandemic.	Cross‐sectional study.	405 nurses from one hospital in China.	Self‐leadership (SL) stimulated thriving at work (TAW) (B 0.659; SE 0.184; *p* < 0.001) and it strengthened more TAW than self‐sacrificial leadership (SSL). In addition, SL weakened the effect of SSL on TAW (B −0.085; SE 0.033; *p* < 0.05). Related to SL, findings supported hypothesis 3 (“During the COVID‐19 pandemic, nurses’ SL has a positive association with TAW”) and hypothesis 4 (“During the COVID‐19 pandemic, nurses’ SL will moderate the relationship between managers’ SSL and nurses’ TAW, such a positive effect will be weakened when SL is high”). Moreover, nurses’ SL was a negative moderator in the indirect relationship between SSL and work well‐being, as well as work–family conflict.	Strengths: ‐ The data collection is described in detail ‐ Large sample size ‐ The study presents bootstrapping results for moderate mediation ‐ The practical implications of the findings are presented concretely and from many perspectives Limitations: ‐ Details of the respondents' exclusion criteria and settings have not been described ‐ Specific crisis context of the COVID‐19 pandemic
Jeon and Park (2020), South Korea	To identify nursing work performance, job satisfaction, and self‐leadership, along with analyze the effects of nursing performance.	Cross‐sectional study.	176 nurses from several hospitals in South Korea.	Nurses assessed their nursing work performance as 3.54 (SD 0.43), job satisfaction as 3.13 (SD 0.45), and self‐leadership as 3.46 (SD 0.40) out of 5. The highest scores of the dimensions of self‐leadership showed self‐compensation, followed by rehearsal, self‐expectation, constructive thinking, self‐criticism, and goal setting. Related to nurses’ demographic characteristics and self‐leadership, the statistically significant difference was shown only in age (F 4.65; *p* = 0.001). The job satisfaction (B 0.17; *p* = 0.001) and self‐leadership (B 0.49; *p* = 0.001) influenced the nursing work performance and the explanation power of them was 40.4%.	Strengths: ‐ The study provides additional knowledge in improving nurses' self‐leadership in hospitals Limitations: ‐ Study subjects, settings, and data collection have been poorly reported ‐ Low sample size ‐ Authors did not highlight any limitations of the study
Kim and Kim (2019), South Korea	To test the effect of self‐efficacy and job embeddedness on self‐leadership.	Cross‐sectional study.	199 nurses from two hospitals in South Korea.	Self‐leadership was higher for nurses over 40 years of age than for younger nurses (F 10.001; *p* < 0.001), as well as when nurses’ academic achievement (F 18.419; *p* < 0.001), clinical experience (F 4.631; *p* = 0.011), and preceptor experience were higher (F 7.237; *p* 0.001). Self‐leadership also was higher for nurses who did not have shift work (*t* −4.591; *p* < 0.001). Self‐efficacy had significant direct and indirect effects on self‐leadership, while job embeddedness had significant direct effects on it. Moreover, job embeddedness had a mediating role in the relationship between self‐efficacy and self‐leadership.	Strengths: ‐ Study subjects, settings, and data collection are described in detail ‐ Structural equation modeling provided Limitations: ‐ The causal relationship between the observed variables was analyzed while the characteristics of participants were not controlled
Kim and Park (2015), South Korea	To identify the factors that affect the innovative behaviors of nurses based on their individual and organizational characteristics.	Cross‐sectional study.	347 nurses from six hospitals in South Korea.	Self‐leadership had a direct positive effect on creative self‐efficacy (β 0.522; *p* < 0.05), on innovative organizational culture (β 0.113; *p* = 0.050), and on individual innovative behaviors (β 0.178; *p* < 0.05). Self‐leadership had an indirect positive effect (β 0.147; *p* < 0.05) on individual innovative behavior through creative self‐efficacy and innovative organizational culture.	Strengths: ‐ The adequacy of the sample size for statistical analysis is justified ‐ Structural equation modeling provided Limitations: ‐ Details of the respondents' inclusion and exclusion criteria, as well as data collection, were not described ‐ The study did not consider staff‐mix level or hospital size
Kim and Sim (2020), South Korea	To establish a model showing a relationship between factors of the nurse's competency (communication ability, self‐leadership, self‐efficacy, and nursing outcomes), and test the model empirically.	Cross‐sectional study.	168 nurses from several hospitals in South Korea.	The average scores of the dimensions of the self‐leadership were Action‐oriented strategies 3.58 (SD 0.64), self‐rewarding strategies 3.78 (SD 0.62), and constructive thinking 2.91 (SD 0.68) out of 5. The relationship between nurses’ self‐leadership and communication ability had a statistically significant effect (β 1.049; *p* < 0.001) but the relationship between self‐leadership and nursing performance did not have. Moreover, the relationship between self‐efficacy and communication ability had a statistically significant effect (β 0.899; *p* < 0.001) and nurses’ communication ability affected nursing performance through self‐efficacy. Finally, the relationship between self‐efficacy and nursing performance had a statistically significant effect (β 0.464; *p* < 0.001).	Strengths: ‐ Pilot study before the actual data collection ‐ The adequacy of the sample size for statistical analysis is justified ‐ Structural equation modeling provided Limitations: ‐ Nurses' individual competencies varied, which limited accurate evaluation of the classification of nursing performance ‐ The response rates varied between the hospitals, which may have biased the results
Kyung and Park (2022), South Korea	To explore nurses' job satisfaction, self‐leadership, and empowerment, along with identifying factors affecting nurses’ job satisfaction.	Cross‐sectional study.	244 nurses from three hospitals in South Korea.	The mean scores for self‐leadership, empowerment, and job satisfaction were 3.38 (SD 0.48), 2.98 (SD 0.51), and 3.08 (SD 0.47) out of 5, respectively. Regarding self‐leadership, religious nurses had significantly higher scores (3.49) than nonreligious ones, as well as nurses with 10 years or more clinical experience (3.54) compared with those with 5–10 years of experience. A strong positive association between self‐leadership and empowerment (*r* 0.485; *p* = 0.001) was found.	Strengths: ‐ The data collection is described in detail ‐ The adequacy of the sample size for statistical analysis is justified Limitations: ‐ Study subjects and settings have not been described in detail ‐ Authors did not highlight any limitations of the study
Mustriwati et al. (2021), Indonesia	To identify the effect of nurses’ self‐leadership and organizational commitment while working at ward for the Covid patients.	Cross‐sectional pilot study.	52 nurses from one hospital in Indonesia.	Findings showed that self‐leadership and organizational commitment concurrently affected the performance of the nurses (*p* = 0.009) and self‐leadership also had a partial effect on the performance of nurses (*p* = 0.044). In addition, organizational commitment had a partial effect on nurse performance (*p* = 0.025).	Strengths: ‐ The study provides additional knowledge in improving nurses' self‐leadership in hospitals Limitations: ‐ Pilot study ‐ Study subjects, settings, and data collection have been poorly reported ‐ Authors did not highlight any limitations of the study
Prinsloo and Jooste (2022), South Africa	To describe the method followed for developing the conceptual framework for how nurses' self‐leadership influenced the functioning of Critical care outreach services (CCOS).	A qualitative study.	Eight focus groups with 57 nurses from one hospital in South Africa.	Nurses experienced self‐leadership strategies such as constructive thought patterns, natural rewards, and behaviors focused on their implementation of CCOS. The theme of nurses being mindful of their self‐leadership exists in the development of self‐motivation and self‐direction. Three categories were identified: self‐motivation, self‐direction through role‐modeling to peers and training.	Strengths: ‐ The study constructs and presents a conceptual framework ‐ Part of larger research on nurses’ self‐leadership ‐ The study has been reported carefully and in detail Limitations: ‐ The data have been collected at one private hospital
Shin and Yeom (2021), South Korea	To explore the effects of the nursing practice environment and self‐leadership of oncology nurses who provide person‐centered care.	Cross‐sectional study.	145 nurses from eight hospitals in South Korea.	The mean scores for nursing practice environment, self‐leadership, and person‐centered care were 3.28 (SD 0.51), 3.28 (SD 0.38), and 3.74 (SD 0.42) of a total of 5, respectively. In the subareas of self‐leadership, the highest score was self‐compensation (M 3.56; SD 0.61), followed by rehearsal (M 3.47; SD 0.62), self‐criticism (M 3.35; SD 0.84), self‐expectation (M 3.19; SD 0.57), constructive thoughts (M 3.11; SD 0.56), and creating goals (M 3.00; SD 0.71). Person‐centered care was correlated with the nursing practice environment (*r* 0.27; *p* < 0.001) and self‐leadership (*r* 0.40; *p* < 0.001), along with the nursing practice environment correlated with self‐leadership (*r* 0.38; *p* < 0.001). Further, controlling for the effects of covariates (salary, total clinical career, and the position of nurses) and nursing practice environment, self‐leadership was a predictor of person‐centered care (β 0.34; *p* < 0.001).	Strengths: ‐ Study subjects, settings, and data collection are described in detail ‐ The adequacy of the sample size for statistical analysis is justified Limitations: ‐ The data were collected from only one nursing specialty: oncology ‐ The instrument used was originally designed for intensive care units
Yu and Ko (2017), South Korea	To identify the effects of self‐leadership and communication competence on the job performance of nurses.	Cross‐sectional study.	211 nurses from six hospitals in South Korea.	The mean scores for self‐leadership and communication competency were 3.70 (SD 0.41) and 3.60 (SD 0.45) out of 5, respectively. In addition, the mean score for job performance was 2.97 (SD 0.28) out of 4. Communication competency and self‐leadership (*r* 0.697; *p* < 0.001), communication competency and job performance (*r* 0.550; *p* < 0.001), and self‐leadership and job performance (*r* 0.599; *p* < 0.001) showed positive correlations.	Strengths: ‐ Study subjects and settings are described in detail ‐ The adequacy of the sample size for statistical analysis is justified ‐ The data collection is described in detail Limitations: ‐ The sample size of the six hospitals was low ‐ Authors did not highlight any limitations of the study

### Data synthesis

Narrative data synthesis was completed for the included studies. A descriptive analysis was performed. The results of the quantitative studies were described qualitatively, and the data synthesis process involved coding, grouping, and combining groups by content into relevant categories and further into descriptive themes (Stern et al., 2020).

## FINDINGS

### Study characteristics

This scoping review included 13 studies conducted in multiple countries, most commonly South Korea (*n* = 7) followed by China (*n* = 2), Indonesia (*n* = 1), Portugal (*n* = 1), Saudi Arabia (*n* = 1), and South Africa (*n* = 1). This review included 12 cross‐sectional studies and one qualitative study and included data collected from 2855 nurses. The number of participants in each study ranged from 52 to 405. Based on the inclusion criteria, only the findings related to nurses working in hospital settings were included.

### Nurses’ self‐leadership in hospital settings

**FIGURE 2:**
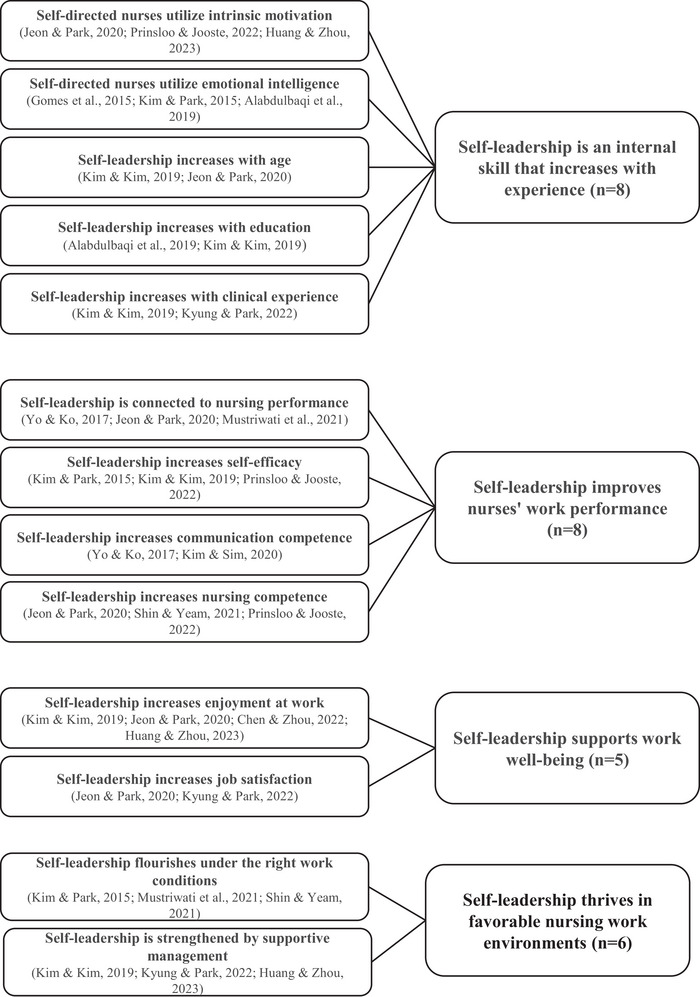
Themes, subthemes, and the number of supporting studies (*n*).

The findings related to nurses’ self‐leadership were combined into four main themes. These themes highlight that nurses’ self‐leadership is an internal skill that increases with experience; self‐leadership improves nurses' work performance; supports work well‐being; and thrives in favorable nursing work environments. The themes, subthemes, and the number of specific studies that support them are illustrated in Figure 2.

### Self‐leadership is an internal skill that increases with experience

Motivation is closely linked to one's self‐leadership skills. Nurses must be motivated to develop their self‐leadership skills. Self‐leadership can also increase the nurse's motivation to develop other skills. Self‐motivated nurses independently decide and act on the basis of their awareness of their competencies (Chen & Zhou, 2022; Jeon & Park, 2020; Prinsloo & Jooste, 2022). Self‐directed nurses also utilize emotional intelligence. Flexible thinking, combined with the ability to control emotions, increases positive and constructive thinking (Alabdulbaqi et al., 2019). Moreover, self‐leadership is related to nurses’ innovative behaviors and ability to encourage new ideas from others (Kim & Park, 2015).

Previous studies have shown that self‐leadership increases with experience and can be seen as learned behavior (Gomes et al., 2015). Nurses’ self‐leadership increases with age (Jeon & Park, 2020; Kim & Kim, 2019). In addition, a positive relationship exists between nurses’ self‐leadership and their level of education and achievements (Alabdulbaqi et al., 2019; Kim & Kim, 2019), as well as between self‐leadership and greater clinical experience. With experience, nurses are ready to have greater responsibility for their tasks (Kim & Kim, 2019; Kyung & Park, 2022).

### Self‐leadership improves nurses' work performance

Self‐leadership has been shown to have a positive effect on nurses’ work performance (Jeon & Park, 2020; Yo & Ko, 2017) and self‐leadership and organizational commitment (Mustriwati et al., 2021). Self‐leadership also increases self‐efficacy. Self‐directed nurses have self‐control and are able to creatively and proactively direct their behaviors to improve patient care (Kim & Kim, 2019; Kim & Park, 2015; Shin & Yeam, 2021; Prinsloo & Jooste, 2022).

Overall, self‐leadership increases nurses’ competence. It allows nurses to enhance their confidence, fulfill their skills, and improve their expertise (Jeon & Park, 2020). Nurses who implement self‐leadership set goals for themselves to develop professionally (Prinsloo & Jooste, 2022). Additionally, previous studies have pointed out that nurses’ self‐leadership is positively related to their ability to communicate (Kim & Sim, 2020; Yo & Ko, 2017).

### Self‐leadership supports work well‐being

Self‐leadership increases enjoyment at work (Jeon & Park, 2020; Kyung & Park, 2022). It is also related to job embeddedness wherein nurses state their willingness to stay in their current job (Kim & Kim, 2019). Moreover, self‐leadership is associated with nurses’ experiences of vitality and success thriving at work and offers them more personal resources for solving problems and acting as required by the situation. Nurses with self‐leadership skills seemed to tolerate uncertainty and stress (Chen & Zhou, 2022; Huang & Zhou, 2023) and reported higher levels of job satisfaction (Jeon & Park, 2020; Kyung & Park, 2022).

### Self‐leadership thrives in favorable nursing work environments

Previous studies have indicated that self‐leadership flourishes under appropriate work conditions. An innovative and modern organizational culture has been found to mediate the relationship between nurses’ self‐leadership and innovative behavior (Kim & Park, 2015). Organizations that enable and support self‐leadership strategies for nurses create a mechanism to deal with different situations in a healthy manner (Kim & Kim, 2019; Mustriwati et al., 2021). Nurses with greater self‐leadership experience their work environments positively (Shin & Yeam, 2021).

Managers’ leadership styles influenced the success of nurses’ self‐leadership. Supportive management influences self‐leadership, and it is positively related to empowerment, where nurses are encouraged to achieve their own goals and have the opportunity to impact organizational goals (Kyung & Park, 2022).

## DISCUSSION

This scoping review summarizes what has been previously described in the literature about the key concepts, characteristics, and factors associated with nurses’ self‐leadership in practice to analyze knowledge gaps (Munn et al, 2022; Peters et al., 2020). A review of the literature showed that self‐leadership in nursing is a little‐studied topic; 1831 articles were included in a comprehensive search of which only 50 full texts were suitable for a closer look. Ultimately, 13 studies met the inclusion criteria. The low number of studies is a surprising finding. Employees in healthcare are expected to lead themselves in ever‐changing environments to meet patients’ needs and achieve organizational goals (Green‐Wilson et al., 2022; Liu et al., 2023). We would have also expected more international knowledge about nurses' self‐leadership. This surprise is also supported by the fact that the phenomenon has been studied outside of nursing for a long time and the concept is generally well known in other fields (Manz, 2015; Neck et al., 2019).

Most of the included studies used a cross‐sectional design (12 of 13), and their reporting styles varied. It would have been beneficial if these cross‐sectional studies had followed the STROBE reporting guidelines. Study subjects, settings, and data collection were often only partly described. Sample sizes were generally low (averaging 233; ranging from 52 to 405), and self‐administered surveys were used, which may not entirely represent the actual practice. However, the instruments were valid and reliable, and the data were analyzed using appropriate statistical methods.

Interpretation of the results of this scoping review must include a recognition that there may be different practice patterns and leadership styles that are more common in some countries than in others. Most previous studies in this scoping review related to nurses' self‐leadership were conducted in South Korea. Using our inclusion criteria, we found no studies from the United States and only one from Europe. The included studies from South Korea did not differ in their research purpose or findings from those conducted elsewhere. All studies strongly emphasized the connection between nurses’ self‐leadership and examination of their work performance, efficiency, or innovation. The connection with job satisfaction has also been examined in studies conducted in different parts of the world. Such a strong geographic focus on South Korea is an interesting finding in relation to nurses' self‐leadership, which could be explained by the organizational expectations and investments related to nurses, as well as management culture. In the United States and Europe, the focus has long been on different leadership styles, such as transformational leadership, and their effects on nursing staff's work performance and job satisfaction, and nursing outcomes (Cummings et al., 2018; Hult et al., 2023).

### Experience of the nurse

Self‐leadership is an internal skill that improves through education, practice, and experience. Nurses use internal resources when drawing on their physical and psychological strengths, including motivation, personal energy, sense of purpose, and spiritual reserves. Some of these traits are likely personality‐related but can also be learned and strengthened through training (Kang et al., 2022). Developing self‐leadership skills should be emphasized in prelicensure academic nursing education programs.

We agree with the conclusions of previous studies that organizations should implement self‐leadership training for nurses to develop their self‐leadership skills. Training can help individuals examine their actions, abandon dysfunctional beliefs, and replace them with more constructive thoughts, as well as identify ineffective behaviors through self‐observation and self‐awareness (Junça‐Silva & Camaz, 2023; Manz, 2015). However, specific details on how to teach self‐leadership behaviors to practicing nurses were not identified in the studies explored within this scoping review. Van Dorssen‐Boog et al. (2021) explored the effect of a self‐leadership intervention in the healthcare industry and found that self‐leadership has positive outcomes related to job performance, engagement, and health. Accordingly, training interventions focused on refining behavior and thought patterns should be offered to nurses to strengthen their internal resources.

### Work performance

Previous studies have highlighted that self‐leadership improves nurses' work performance, which has been explained by behavior‐focused strategies. Self‐leadership generally has a strong relationship with innovation and creativity (Knotts et al., 2021; Neck et al., 2019). Nurses must take responsibility for their work to provide smooth, efficient, and safe nursing care. Using behavior‐focused strategies, such as negative thought‐stopping and focusing on positive experiences and interactions, nurses become better problem solvers and act in the best interests of their patients (Prinsloo & Jooste, 2022). Our review indicated that nurses’ communication skills were related to self‐leadership. Effective communication is central to intra‐ and interprofessional cooperation and ultimately leads to safer and better‐quality patient care and the overall development of nurses' self‐leadership.

### Work well‐being

Satisfied nurses with a strong sense of well‐being provide safe and high‐quality patient care (Labrague, 2024). Well‐being has also been emphasized in studies outside nursing (Junça‐Silva & Camaz, 2023; Knotts et al., 2021; Müller & Niessen, 2019). Job satisfaction and well‐being are often explained by external factors such as management and organizational structure. While acceptable salary or pay and formal recognition strategies are important, our review emphasizes the value of supporting nurses’ self‐leadership skills. Individuals with greater self‐leadership skills look for opportunities to make their work more pleasant so that the work itself becomes naturally rewarding and they do not spend wasted energy on things beyond their control (Neck et al., 2019). When individuals feel free to practice autonomy in their workplaces, these internal rewards allow them to increase their work engagement and health (Inam et al., 2023; Van Dorssen‐Boog et al., 2020).

### Work environment

Nurses with greater self‐leadership usually experience a positive work environment (Shin & Yeam, 2021). Self‐leadership thrives in a favorable nursing work environment supported by innovative organizational cultures (Kim & Park, 2015). To improve nurses' self‐leadership skills, nurses need to be offered opportunities to participate and influence at different levels of the organization. Many nurses, however, have limited opportunities to influence beyond the unit level (Pursio et al., 2023). Recent research on shared governance and nurses’ professional autonomy highlights that their expertise should be identified and maximized in healthcare organizations (Porter‐O'Grady & Clavelle, 2021; Pursio et al., 2024). Professional nurses have the responsibility to develop their competence and expertise. Nurse leaders can assist them in strengthening their self‐leadership skills through reflection, training, and regular feedback.

### Nurse self‐leadership compared with other professionals

Our findings on nurses’ self‐leadership revealed the same themes previously described in other fields. Innovative work performance, including creative problem‐solving, competence, and commitment to work, is the main focus of self‐leadership studies (Kang & Song, 2022; Knotts et al., 2021). Nursing‐specific connections have not been highlighted in previous studies. Research on nurses' self‐leadership appears to be guided by three self‐leadership strategies, including behavioral‐, natural reward‐, and constructive thought‐strategies, studied with commonly used instruments (e.g., Houghton & Neck, 2002) used for many fields. This enables a comparison of self‐leadership of different professionals, which is a key topic for further research. Other important suggestions for future research are to explore the effect of a specific intervention on nurses' self‐leadership, the connection between nurses’ self‐leadership to nursing outcomes, and what kinds of self‐leadership skills are emphasized in different nursing work environments. As the concept is still somewhat unclear in nursing, qualitative research is needed.

### Limitations

This scoping review has a few limitations. While we conducted an extensive literature search in collaboration with an information specialist, scoping reviews generally include evidence from both previous research and nonresearch sources (Peters et al., 2020). Our search strategy focused on four databases, and reports in the gray literature were not included. Several studies did not report the methods in detail; thus, methodological weaknesses may have occurred.

More than half of the studies were conducted in South Korea and may reflect nursing practice in this country, therefore, limiting the global generalizability of the results of this scoping review. Several quantitative studies provide information on similar concepts related to nurses’ self‐leadership (e.g., self‐motivation and internal control). Causality cannot be determined with cross‐sectional designs. Only one qualitative study was included in this review; more qualitative studies would have added value to the description of self‐leadership as a phenomenon in the context of professional nursing. There were also limited examples of how to support or develop self‐leadership behaviors through training.

## IMPLICATIONS FOR NURSING EDUCATION, PRACTICE AND POLICY

The international nursing profession will be strengthened by nurses who develop self‐leadership behaviors throughout each developmental stage of their careers following actionable recommendations for nursing education, practice, and policy. Prelicensure nursing students should be guided to participate in self‐reflection of intrinsic motivational behaviors, develop assertive communication strategies through simulation, and engage in evidence‐based practice activities grounded in a spirit of inquiry. Nursing faculty can help students recognize growth in self‐leadership behaviors in clinical settings through formative and summative evaluations. Transition to practice or nurse residency programs should be offered to all newly licensed nurses. Nurse managers should provide frequent feedback related to competency development in clinical problem‐solving, interprofessional communication, and other leadership behaviors. Hospital leaders should develop policies that encourage nurse self‐leadership behaviors by providing forums and structures such as shared governance models and programs that promote healthy work environments.

## CONCLUSION

Self‐leadership has a positive connection to nurses’ work performance and well‐being and is critical to quality patient care. While self‐leadership is a little‐studied topic among nurses, four primary themes emerged in this scoping review: (1) self‐leadership is an internal skill that increases with experience; (2) self‐leadership improves nurses' work performance; (3) self‐leadership supports work well‐being; and (4) self‐leadership thrives in favorable nursing work environments. Self‐leadership, according to these themes, can be encouraged through each developmental stage of a nurse's career. Nursing students should be introduced to self‐leadership skill development in their basic nursing education programs, and nurses should be offered continuing education opportunities to develop self‐leadership skills throughout their working careers. Additional studies are necessary to explain self‐leadership in the context of professional nursing and offer recommendations for how to strengthen nurses’ self‐leadership skills through basic training in prelicensure programs and continuing education for practicing nurses.

## AUTHOR CONTRIBUTIONS

*Study design*: Katja Pursio, Tarja Kvist, Päivi Kankkunen, and Laura A. Fennimore. *Database search*: Katja Pursio. *Data analysis and synthesis*: Katja Pursio, Tarja Kvist, Päivi Kankkunen, and Laura A. Fennimore. *Study supervision*: Tarja Kvist, Päivi Kankkunen, and Laura A. Fennimore. *Tables and figures*: Katja Pursio. *Manuscript writing (original draft)*: Katja Pursio, Tarja Kvist, Päivi Kankkunen, and Laura A. Fennimore. *Critical revision*: Katja Pursio, Tarja Kvist, Päivi Kankkunen, and Laura A. Fennimore.

## CONFLICT OF INTEREST STATEMENT

The authors declare no conflict of interest.

## FUNDING INFORMATION

This research received no specific grant from any funding agency in the public, commercial, or not‐for‐profit sectors.

## ETHICS STATEMENT

This study does not involve human participants; and therefore, it was not necessary to obtain ethical approval.

## Supporting information



Supporting Information
