# Supplementary material for: Self‐leadership and why it matters to nurses: A scoping review
Source: Int Nurs Rev. 2025 Mar 5;72(1):e70014. doi: 10.1111/inr.70014 (PMC11881033; doi:10.1111/inr.70014)
Supplement: Supplementary file 1 — Supporting Information [file INR-72-0-s001.docx]

Supplementary file 1. Search strategy

| **Database** | **Search terms** | **Limits** | **Records** |
| --- | --- | --- | --- |
| PubMed | nurse AND (self-leadership OR “self leadership” OR self-concept OR “self concept” OR self-regulation OR “self regulation” OR self-actualization OR “self actualization” OR self-determination OR “self determination”) AND hospital | English language  Published between 2013–2023 | 681 |
| CINAHL |  |  | 398 |
| Scopus |  |  | 1103 |
| PsycINFO |  |  | 204 |
